# A Narrative Review of Invasive Candidiasis in the Intensive Care Unit

**DOI:** 10.1177/29768675241304684

**Published:** 2024-12-12

**Authors:** Elnè Noppè, Julian Robert Paul Eloff, Sean Keane, Ignacio Martin-Loeches

**Affiliations:** Department of Intensive Care Medicine, Multidisciplinary Intensive Care Research Organization, 58024 (MICRO), St James' Hospital, Dublin, Ireland; Department of Anaesthesia, 73003 Port Shepstone Regional Hospital, Port Shepstone, South Africa; Department of Intensive Care Medicine, Multidisciplinary Intensive Care Research Organization, 58024 (MICRO), St James' Hospital, Dublin, Ireland; Department of Intensive Care Medicine, Multidisciplinary Intensive Care Research Organization, 58024 (MICRO), St James' Hospital, Dublin, Ireland

**Keywords:** Intensive care unit, invasive candidiasis, antifungal therapy, critical care, medicine, infection, sepsis

## Abstract

*Candida* species is the most common cause of invasive fungal infection in the critically ill population admitted to the intensive care unit (ICU). Numerous risk factors for developing invasive candidiasis (IC) have been identified, and some, like the breach of protective barriers, abound within the ICU. Given that IC carries a significant mortality, morbidity, and healthcare cost burden, early diagnosis and treatment have become an essential topic of discussion. Several expert panels and task forces have been established to provide clear guidance on the management of IC. Unfortunately, IC remains a diagnostic and therapeutic challenge attributable to the changing fungal ecology of *Candida* species and the emergence of multidrug-resistant strains. This narrative review will focus on the following: (1) the incidence, outcomes, and changing epidemiology of IC globally; (2) the risk factors for developing IC; (3) IC risk stratification tools and their appropriate use; (4) diagnosis of IC; and (5) therapeutic agents and regimens.

## Plain Language Summary Title

A review of severe Candida infection in patients admitted to the Intensive Care Unit Plain Language Summary:

Candida infections are the most common type of serious fungal infections that affect patients in Intensive Care Units (ICUs). There are many known risk factors for developing these infections, especially in ICU patients who often experience situations like damage to protective barriers in the body. Since these infections can lead to death, serious illness, and higher healthcare costs, itâ€™s important to diagnose and treat them as early as possible. However, diagnosing and treating invasive Candida infections is still very difficult. This is because the types of Candida fungi are changing, and some strains are becoming resistant to multiple antifungal medications. The goal of this review is to help improve understanding and management of these serious infections in ICU settings.

## Introduction

*Candida* is a normal part of human flora, most frequently isolated from the gut.^1^ Invasive candidiasis (IC) develops when disruptions in immune barriers allow *Candida* to translocate into the bloodstream (candidemia) or result in deep-seated localized infections.^2^ IC is particularly prevalent in intensive care units (ICUs), where its incidence is 10 times greater than in medical and surgical wards, accounting for between 70% and 90% of all ICU fungal infections.^1^ This is partly due to an ageing patient population, severe acute illness, invasive procedures, use of immunosuppressants, and broad-spectrum antimicrobials.^1^ IC encompasses both candidemia and deep-seated *Candida* infection with or without the associated bloodstream infection, with intra-abdominal candidiasis (IAC) being the most common deep-seated infection.^1^ We completed a MEDLINE search for English articles published between 1998 and 2024 using the terms “Invasive Candidiasis,” “Candidaemia,” “Epidemiology,” “Diagnosis,” “Biomarkers,” and “Treatment” and also reviewed the reference lists of articles identified in this search. We aim to produce an up-to-date and concise overview of IC in the ICU.

## Epidemiology

The epidemiology of *Candida* infections in the ICU involves a complex interplay of patient risk factors and antifungal resistance. The observed rise in the prevalence of yeast infections in ICUs and the associated mortality rate from candidemia could be influenced by several factors.^3,4^ Therefore, a comprehensive analysis of IC incidence over several years and across various countries is necessary to discern whether the observed increase is due to changes in diagnostic practices or a *bona fide* increase in prevalence and mortality rates. In this context, epidemiological surveillance remains essential.

While numerous registries exist for ICUs, their global representation is limited. Additionally, regions with a low to lower-middle income per capita need to be more represented. It's crucial to note that an isolate does not necessarily equate to an infection, as *Candida* species are a frequent commensal fungus that colonize the oropharyngeal cavity, gastrointestinal and vaginal tract without causing disease.^5^ In Europe, the incidence of IC was 7.0 cases per 1000 ICU admissions, with a crude mortality rate of approximately 40%.^6^ The incidence and distribution of *Candida* species varies within different regions with a reported higher incidence for IC in Southern Europe than in Northern or Western Europe.^3,6,7^ *C. albicans* remains the leading cause of IC in Europe, the United States, and Australia, followed by *C. glabrata*.^7–9^ Interestingly, In Asia and South America, non-*albicans Candida* (naC) is dominant, with *C. parapsilosis* and *C. tropicalis* being the most frequently isolated, respectively.^10^ Despite its vast population and geographic diversity, data on IC in Africa remain limited. A comprehensive review of IC cases across Africa reported only 18 293 cases over 45 years. Of these, *C. albicans* was the most common species (32.6%), followed by *C. parapsilosis* (30.4%) and the emerging *C. auris* (7.8%).^11^

There is a trend of increasing naC prevalence in Europe and America, but not in Asia and South America (which already have a higher prevalence of naC).^10^ The shift towards naC is concerning as many of these *Candida* species have intrinsic azole resistance (*C. glabrata* and *C. krusei*), are adept at forming biofilms (*C. parapsilosis*), and easily develop resistance (*C. glabrata*).^12,13^

A microcosm of epidemiological shift can be seen with *C. parapsilosis*. Although initially considered fully susceptible to fluconazole, the emergence of fluconazole-resistant *C. parapsilosis* has been reported in a growing number of countries.^13^ In Africa, the emergence of azole resistance has propelled *C. parapsilosis* to the second most common cause of IC.^11^ Since 2018, these resistant strains have expanded and outcompeted fluconazole-sensitive strains in Southern Europe, leading to major outbreaks in Spain, Greece, Italy, and Turkey.^13^

The growing concern of azole resistance in naC and reported echinocandin resistance is further complicated by the emergence of *C. auris*.^4^ This multidrug-resistant yeast has appeared almost simultaneously in various locations worldwide. It poses an additional challenge in the diagnosis and management of IC, and has been linked with outbreaks globally, particularly in ICUs.^3,4,14^ Identifying this species requires molecular methods and is often misidentified. A retrospective review of the SENTRY isolate collection found no *C. auris* isolates before 2009, indicating that the pathogen was genuinely rare and not merely misidentified in the past.^15^ The rapid emergence may partly be due to the increased availability of antifungal agents and the resulting selection pressure. *C. auris* appears to be inherently resistant to azoles, with variable susceptibility to amphotericin B and echinocandins. This is evident in South Africa, where a unique clade of *C. auris* has emerged in hospitals.^16^ This is believed to have occurred due to the indiscriminate use of fluconazole as prophylaxis and treatment as well as suboptimal adherence to infection control practices.

Current evidence indicates that major risk factors for IC, such as intestinal mucosal barrier damage, abdominal surgery, pancreatitis, and dysbiosis of resident microbiota, are more common in surgical ICUs.^1,2^ In contrast, patients in medical ICUs are also at risk for IC due to factors such as the growing use of antineoplastic and immunosuppressive therapies, an increased number of hematopoietic stem cell transplants, and the widespread use of broad-spectrum antibiotics.^17^

The limitations identified in the studies on the prevalence of IC in ICUs include challenges in ascertaining the true incidence of candidemia due to the use of different denominators such as indwelling catheter duration, bed days, admissions, discharges, and patient days.

## Risk Factors and Stratification

Given the time-sensitive nature of initiating treatment for IC to prevent significant morbidity and mortality, early suspicion and diagnosis are essential. Thomas-Rüddel et al^18^ conducted a meta-analysis which identified broad-spectrum antimicrobials, blood transfusion, *Candida* colonization, the presence of a central venous catheter, and total parenteral nutrition (TPN) as the factors associated with the highest risk of developing IC. It is thus not surprising that these factors are often included in risk stratification tools, the most widely known being the *Candida* Colonization Index, the *Candida* Score, and the Ostrosky-Zeichner Clinical Prediction Rule. Additionally, mechanical ventilation, renal replacement therapy, and other breaches of protective barriers such as extracorporeal membrane oxygenation also played a major role. Finally, there are risk factors that are more difficult to define such as “prolonged” ICU stay and genetic polymorphisms that have also come to light.^7,18^ The development of IC is ultimately dependent on the incidence and distribution of *Candida* species in each unit, as well as the summation and interplay of these risk factors.^7,18,19^

The utility of risk stratification tools for guiding the diagnosis and initiation of antifungal therapy remains contentious. Numerous scoring systems have been developed to identify patients who may benefit from early empiric antifungal treatment; however, these tools generally demonstrate low to moderate positive predictive values (PPVs) of 10% to 66% and high negative predictive values (NPVs), above 90% (Table 1).^20–25,26^ A 2022 systematic review by Giacobbe et al^24^ evaluated the diagnostic accuracy of these tools, including modified versions, across 16 studies and corroborates this, even among symptomatic patients. Consequently, expert recommendation is to use these tools primarily to rule out patients at low risk of IC and for who empiric antifungal therapy should not be a consideration, while identifying those who require further diagnostic evaluation.^25^

**Table 1.** Risk stratification tools for IC.

Risk stratification tool	Performance	Additional information	References
Colonization Index			
*Candida* Colonization Index	PPV 66% NPV 100%	Requires surveillance cultures	^22, 23^
Clinical prediction scores			
*Candida* Score	Sensitivity 81% Specificity 74% PPV 16% NPV 98%	Improved specificity and PPV in combination with BDG	^23, 24, 25^
Ostrosky-Zeichner Clinical Prediction Rule	Sensitivity 50% Specificity 83% PPV 10% NPV 97%		^26^

## Diagnosis

Given that the risk stratification tools have more value as a “rule out” than a “rule in” predictor, it stands to reason that the development of rapid turnover, sensitive, and specific laboratory tests are of paramount importance in patients where IC cannot be ruled out. Unfortunately, no such test yet exists. Conventional culture-based methods remain the gold standard for diagnosing IC, despite limitations such as delayed results, low sensitivity (∼75% in bloodstream infections, ∼5%-20% in IAC), and challenges with sterile site sampling for deep-seated infections.^7,25^ These limitations underscore the importance of culture adjuncts. Mass spectrometry, especially matrix-assisted laser desorption/ionization time-of-flight, is cost-effective and has the ability to rapidly identify *Candida*, including the often misdiagnosed *C. auris*. It also holds promise for detecting antifungal resistance.^25,27,28^ The Peptide Nucleic Acid in Situ Hybridisation Yeast Traffic Light (PNA-FISH YTL) system provides species identification and azole sensitivity grouping within an hour, but its high cost and inability to distinguish between *C. albicans* and *C. parapsilosis*, or between the azole-resistant *C. glabrata* and *C. krusei*, limit its use.^26,29–31^

Given the delay in turnaround time for culture-based diagnosis and that cultures are negative in approximately 50% of IC, non-culture-based tests (NCBT) are important IC diagnostic tools.^32^ NCBT have a rapid turnover time, which may aid in decreasing time to treatment initiation, thus guiding empiric and pre-emptive antifungal treatment, and could possibly be useful in advising treatment duration and monitoring clinical improvement, given that NCBT remains positive for longer than cultures.^7,25^ However, clinician interpretation is crucial, as pretest probability and diagnostic thresholds affect their interpretation.^7,25,33^ Most NCBT display a low PPV, but have high NPV (see Table 2).^7,25,33^ Of the NCBT, the serological test 1,3-β-D-Glucan (BDG) is widely used and validated, and is included in the European Organization for Research and Treatment of Cancer/Mycosis Study Group diagnostic criteria for invasive fungal infection. It is a cell wall constituent of most pathogenic fungi (ie, *Candida*, *Aspergillus*, and *Pneumocystis*), and thus inherently lacks specificity. Its performance is improved when 2 positive results are obtained or used in conjunction with *Candida* risk prediction models, PCT or other fungal biomarkers.^7,25,33,34^ Importantly, the CandiSep trial found no 28-day mortality benefit with the use of BDG for antifungal treatment guidance in ICU patients with risk factors for IC.^35^ Additional considerations include the high false positive rate associated with BDG due a range of factors including (but not limited to) hemodialysis, certain B-lactam antibiotics, human blood products, and TPN, all of which are frequently present in the ICU population.^33^

**Table 2.** Overview of non-culture-based tests.

Non-culture-based tests	Description	Performance	Additional information	References
Polymerase chain reaction				
Multiplex *Candida* real time PCR panel	DNA segment detection and amplification	Sensitivity: 73% to 95% Specificity: 92% to 95%	Not FDA approved Still requires validation in large multi-center studies Performance in IC without candidemia uncertain Performance heterogeneity depending on assay, sample type and study design	^25, 36, 40^
Miniaturized magnetic resonance-based technology				
T2 *Candida*	DNA amplified by PCR, the amplified product is then identified by agglomeration of supermagnetic particles and T2 magnetic resonance	Sensitivity: ∼91% Specificity: ∼94%	Capable of identifying 5 most common *Candida* spp: *Candida*: *albicans*, *glabrata*, *parapsilosis*, *tropicalis*, and *krusei* FDA approved	^29, 36, 41^
Serological tests				
Mannan antigen detection	Mannan is a fungal cell wall constituent	Sensitivity: 58% Specificity: 93%	Combined mannan and anti-mannan sensitivity: 83% and specificity: 86% Not FDA approved	^42^
Anti-mannan IgG antibody detection	Mannan is a fungal cell wall constituent	Sensitivity: 59% Specificity: 83%	Combined mannan and anti-mannan sensitivity: 83% and specificity: 86% Not FDA approved	^43^
CAGTA	Detects antibodies against a hyphal protein expressed by *Candida* spp	Sensitivity: 59% to 73% Specificity: 58% to 88%	Not FDA approved	^36, 43^
BDG	Cell wall constituent of most fungal cell walls	Sensitivity: 77%-81% Specificity: 60% to 85%	See text body for additional information	^44, 45, 46^

Polymerase chain reaction (PCR)-based tests, despite lacking FDA approval, are widely available and can quickly identify common IC-associated *Candida* species.^25,33^ The T2 Magnetic Resonance *Candida* assay (T2 *Candida*) is an FDA approved, fully automated, novel detection technique that offers rapid (3-5 h) and highly sensitive (>90%) detection of *Candida* species in patients with low fungal burdens.^33,36^ Other serological biomarkers that are widely used across Europe for the early detection of IC include mannan antigens, anti-mannan IgG antibodies, and *Candida* species germ tube antibodies (CAGTA), all of which exhibit low sensitivity and specificity^33,37,38^ (Table 2). Presepsin is a new and accurate sepsis biomarker, with a turnaround time of 15 min.^39^ Its use in combination with BDG holds promise for distinguishing between IC and colonization.^40^

IAC diagnosis is especially challenging, as candidemia occurs in only 4% to 14% of patients.^41^ Confirming IAC without candidemia requires a culture from a specimen obtained from a normally sterile site via sterile procedure, and does not differentiate between *Candida* colonization and infection. NCBT may help provide clarity in these cases.^10,42^ A recent study suggests that low BDG concentrations in peritoneal fluid could potentially rule out IAC.^43^ Given the challenges in diagnosing IAC, including the aforementioned limitations of blood cultures and its nonspecific clinical presentation, clinicians must remain mindful of the low sensitivity of traditional tests and by extension, the underreporting in surveillance studies.

As alluded to, the NPV and PPV of both NCBT and risk stratification scores may be improved through combinations of 2 different laboratory tests or by pairing one laboratory test with a clinical score.^7,24,25^ Preliminary data from several studies suggest that these combinations may enhance diagnostic accuracy for IC, particularly by improving PPV when both tests indicate IC and NPV when neither test does.^24^

## Treatment

Antifungal stewardship represents a coordinated, strategic effort to optimize the use of antifungal medications. This includes implementing guidelines for appropriate antifungal therapy, utilizing diagnostic tools to tailor treatment, promoting de-escalation from broad-spectrum to targeted therapy when possible, and monitoring the use of antifungal agents to ensure their judicious and effective utilization. The goal is 4-fold: to reduce unnecessary usage, minimize adverse effects, maximize cure rates, and reduce resistance emergence.

Currently, 4 classes of antifungals are available for systemic treatment of *Candida* species infections in clinical practice.

- Azoles (fluconazole, itraconazole, isavuconazole, posaconazole, and voriconazole)
- Polyenes (conventional amphotericin B and its lipid formulations)
- Echinocandins (anidulafungin, caspofungin, and micafungin)
- Pyrimidine analog (flucytosine)

Azoles work by inhibiting the synthesis of ergosterol, an essential component of the fungal cell membrane. This disruption leads to cell death. Azoles are fungistatic against *Candida* species.^44^ The effectiveness of azoles is concentration- and time-dependent, with a prolonged post-antifungal effect.^25^ Azoles have variable activity against biofilms, with some studies suggesting that they may have only limited effectiveness in eradicating fungal biofilms due to altered metabolic states and reduced susceptibility of biofilm-encased organisms.^27^ Echinocandins target the fungal cell wall by inhibiting glucan synthesis, showing fungicidal activity against most *Candida* species, including biofilm-forming and azole-resistant strains.^7,27^ Historically, Amphotericin B was used for severe fungal infections due to its broad spectrum of activity and fungicidal properties against most *Candida* species. However, its association with nephrotoxicity has led to a decrease in its use. More recently, better-tolerated formulations of amphotericin B, such as liposomal amphotericin B (L-AmB), amphotericin B lipid complex, and amphotericin B colloidal dispersion, have been developed, providing alternatives with reduced toxicity profiles but similar clinical efficacy.^45^ In cases of multidrug-resistant *Candida* infections or for better tissue penetration, amphotericin B may still be considered.^25^ Flucytosine, a synthetic compound, is converted into 5-fluorouracil after being taken up by susceptible fungal cells. This conversion inhibits fungal nucleic acid synthesis. Flucytosine is fungistatic and resistance frequently develops, so it should not be used as monotherapy. It should be used only as an adjunct to other drugs to enhance efficacy and prevent the development of resistance.^46,47^

Pharmacokinetic variability is prevalent in critically ill patients due to factors such as capillary leak, altered protein binding, augmented renal clearance, extracorporeal circuits, and end-organ dysfunction. Consequently, standard dosing regimens may be inadequate, necessitating dosage adjustments, and lead to unpredictable clinical outcomes. In such cases, therapeutic drug monitoring (TDM)-guided dosing is a safe and effective way to ensure that all critically ill patients achieve therapeutic antimicrobial exposures. TDM is most helpful for mold-active azoles (eg, voriconazole) and flucytosine as echinocandins and polyenes appear to be less affected by pharmacokinetic changes in critical illness.^48,49^ While TDM may be used in selected cases, its routine use is not recommended in current guidelines and its precise role in the treatment of IC remains to be defined.

It is essential to acknowledge that the majority of clinical trials and therefore treatment guidelines are biased toward patients with candidemia. This stems from the relative ease of identifying candidemia compared to other forms of IC. Therefore, these patients are more likely to be enrolled in clinical studies than patients with deep-seated candidiasis. Other IC conditions for which higher doses of echinocandins are suggested are endocarditis, IAC, and esophageal candidiasis.^50,51^

Echinocandins demonstrate limited peritoneal penetration, which is further reduced in critically ill patients. For instance, anidulafungin concentrations in these patients have been reported to be approximately 30% of those observed in healthy individuals, increasing the risk of insufficient drug exposure at the site of infection. Therefore, it has been suggested that L-AmB be used for IAC.^50,52,53^ L-AmB is less affected by pharmacokinetic disturbances and has powerful action against biofilm formation, although strong evidence about its peritoneal penetration is lacking.^52^ The use of L-AmB is supported in cases involving hard-to-reach sites such as the central nervous system, and the eyes but, notably, not the urinary tract.^27,50^ Unfortunately cost considerations present a significant barrier to the use of L-AmB.

Furthermore, combining antifungal therapy with adequate source control measures such as removing indwelling catheters, appropriate drainage, and surgical control is essential.^25^ Removal of indwelling catheters is nuanced, but recommendations advocate for the removal of indwelling catheters as early as possible when the source is presumed to be the catheter and the catheter can be removed safely, but this decision should be individualized for each patient.^54^

*Prophylactic therapy*: Patients with no evidence of current infection, clinical or biomarker, but who are at high risk of developing IC due to risk factors. Despite reductions in IC, the mortality benefit remains to be proven. The current European Society of Intensive Care Medicine and European Society of Clinical Microbiology and Infectious Diseases (ESICM/ESCMID) recommendation is against the use of antifungal prophylaxis in any critically ill non-neutropenic patients.^25,55^ The Infectious Diseases Society of America (IDSA) guidelines do allow for antifungal prophylaxis in adult ICUs with a high rate of IC (>5%) in “high-risk” patients.^50^ However, the guidelines do not explicitly define “high-risk” patients, and institutional protocols will differ regarding eligibility for prophylactic therapy. In alignment with the current ESICM/ESCMID recommendations, this author group advocates for prophylactic antifungal treatment exclusively in neutropenic patients.^25^

*Empiric therapy*: Is the use of antifungal therapies in patients with signs and symptoms of infection, plus risk factors for IC, without proof of fungal infection. Echinocandins are the preferred choice for initial empiric antifungal treatment in critically ill patients. This preference is due to their wide range of activity, which includes naC, as well as a safety profile and a drug interaction profile that is more favorable than other antifungal agents.^27,55,56–59^

**Figure 1.**
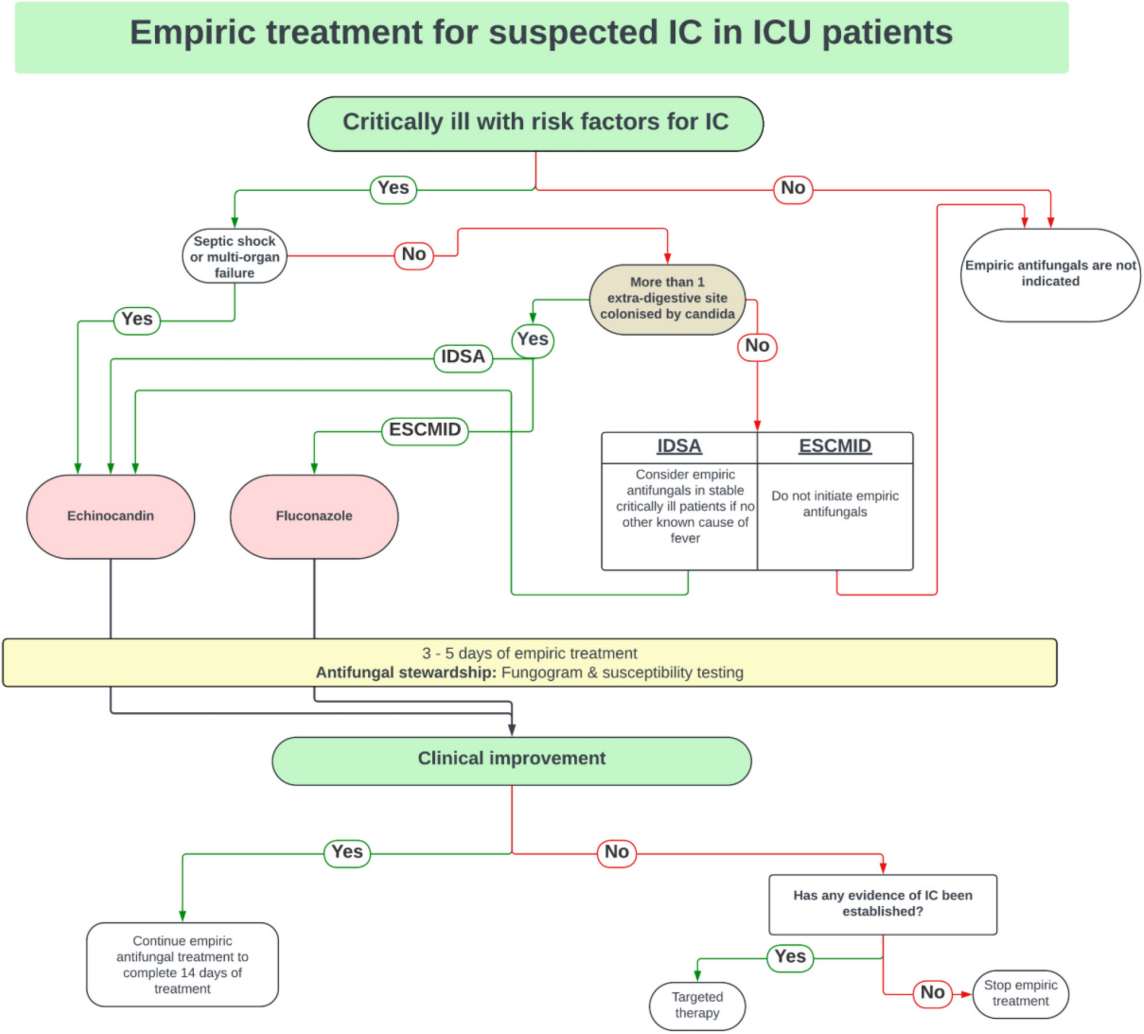
Summary of IDSA and ESICM/ESCMID approach to empiric antifungal therapy.

The key to the successful use of empiric therapy is selecting the appropriate target patient population as highlighted by the EMPIRICUS trial. They demonstrated a reduced incidence of invasive fungal infections in the empiric treatment group, which did not translate into a survival benefit. Empiric therapy should be initiated for patients with septic shock and multi-organ failure, who have more than 1 extra-digestive site colonized by *Candida* species—this is in accord with both IDSA and ESICM/ESCMID guidelines.^25,50,58^ *Figure 1* summarizes the ESICM/ESCMID and IDSA empiric antifungal guidelines, highlighting their subtle differences.

*Directed/targeted therapies*: Are based on microbiological confirmation of an invasive infection due to *Candida*. Prompt and appropriate treatment to prevent complications and improve patient outcomes should be initiated in patients with proven candidemia. ESICM/ESCMID suggests that fluconazole be considered in selected stable patients with IC, without prior azole exposure.^25^ Further, those patients treated with fluconazole should receive a loading dose and a weight-based dosing regimen. However, due to its limited spectrum of activity and lack of fungicidal activity, current IDSA guidelines recommend echinocandins as first-line therapy.^50^ IDSA considers fluconazole an alternative to echinocandins if fluconazole-resistant species are unlikely, irrespective of prior exposure. ESICM/ESCMID recommends echinocandins as the first treatment option in patients with septic shock and IC. For cases involving foreign bodies, echinocandins are preferred due to their enhanced activity against biofilm.^25,50^

**Figure 2.**
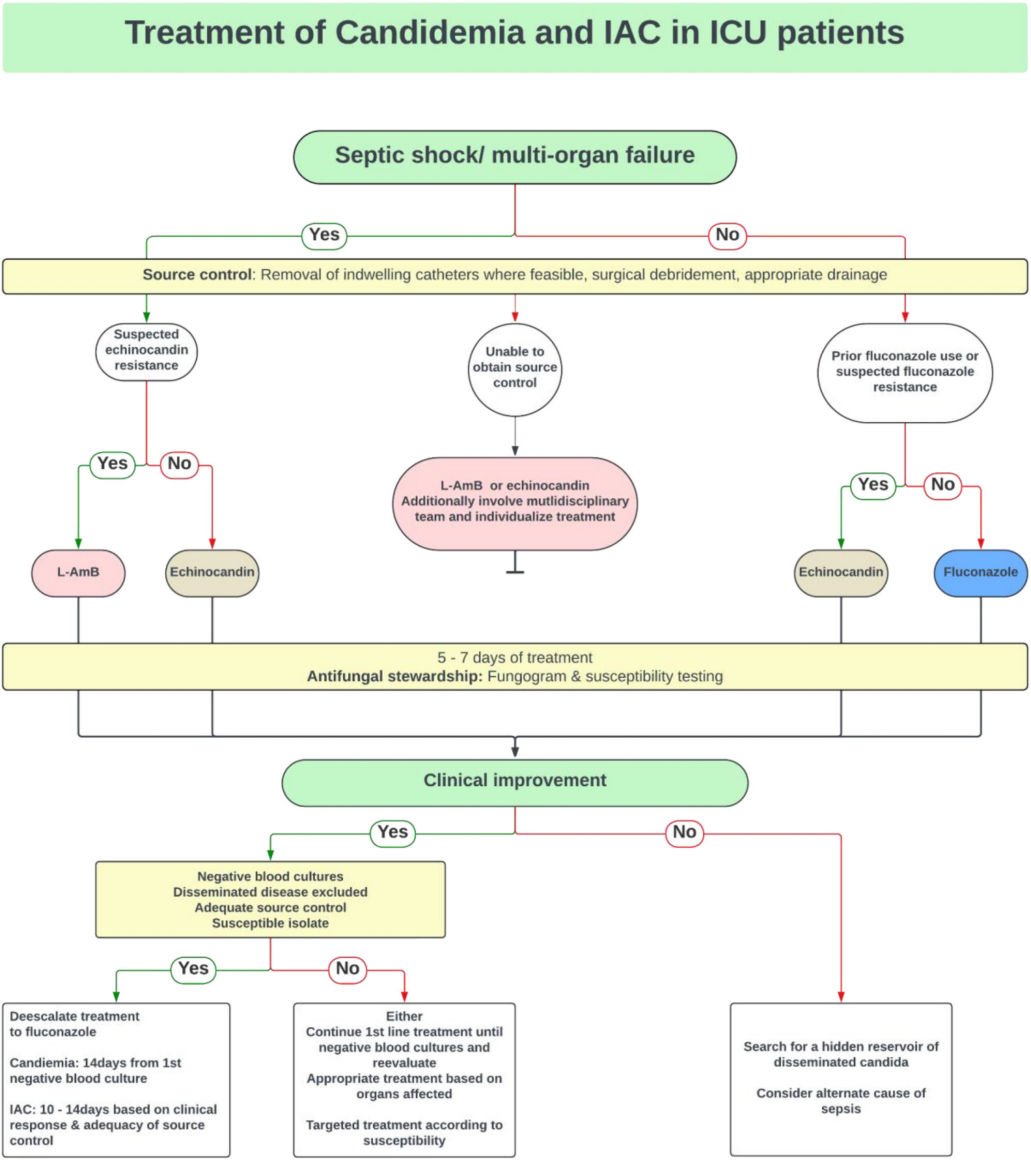
Summary of the treatment of IAC and candidemia in non-neutropenic ICU patients.

In cases of suspected echinocandin resistance, both guidelines suggest using liposomal amphotericin B (L-AmB). For patients unable to remove indwelling catheters, ESICM/ESCMID supports using L-AmB or echinocandins, while IDSA specifically recommends L-AmB for non-removable intracardiac devices. Both guidelines advocate de-escalation to fluconazole, with IDSA suggesting de-escalation within 5 to 7 days, while ESICM/ESCMID does not specify an exact timeframe. The duration of therapy is consistent between guidelines, requiring 2 weeks of treatment after negative blood cultures. For IAC without positive blood cultures, ESICM/ESCMID recommends 10 to 14 days of treatment, whereas IDSA advises a 2-week course, depending on source control and clinical response.^25,50^ There is no consensus on whether blood cultures should be taken daily or every other day until negative growth, but current guidelines accept both approaches.^25,50^ The authors have provided a summary of the ESICM/ESCMID and IDSA guidelines for treating IC in non-neutropenic patients, as outlined in *Figure 2*.

In cases where blood cultures are negative and disseminated disease has been excluded, a treatment period of 10 to 14 days is deemed acceptable. This recommendation is based on clinical evidence suggesting that shorter durations within this range are effective for resolving the infection when blood cultures are negative from the outset.^25^

De-escalation has been proposed with a transition from an echinocandin (or amphotericin B) to an azole in clinically stable patients, with isolates that are susceptible to the azole and have negative repeat blood cultures (in the case of candidemia) following initiation of antifungal therapy. This transition may be safely performed within 5 days after initiation of antifungal therapy.^60^

## Conclusion

Despite ongoing research and expert advisory committees, IC remains a diagnostic and therapeutic challenge in the ICU, with significant morbidity, mortality, and healthcare cost consequences. The incidence and mortality rate of IC continue to rise, reflecting the changing epidemiology of the disease. However, the lack of consistent terminology and denominators complicates accurately determining the incidence and prevalence of IC, impacting research and the development and implementation of guidelines. Risk prediction models are valuable tools for identifying patients needing further IC evaluation, and many models require additional validation. No perfect NCBT exists yet; these tests should be used alongside risk stratification and culture-based testing to inform clinical decisions about initiating and discontinuing antifungal therapy. Although no survival benefit has been conclusively demonstrated with empiric or prophylactic antifungal therapy, empiric therapy remains recommended for select groups of critically ill patients. In the writing of this manuscript, the authors have identified the following areas that necessitate further research and development:

- Consistent terminology and denominators in order to accurately describe IC and determine its incidence in ICU.
- Antifungal stewardship programs that work in parallel with existing antibiotic stewardship programs.
- Risk stratification tools with high PPV to identify which patients would benefit from empiric antifungal therapy.
- Risk stratification tools that take into consideration factors like patient population diversity, regional fungal epidemiology, and healthcare resource availability to ensure universal applicability across diverse regions and patient demographics.
- A cheap and rapid turnover diagnostic test with a sensitivity and specificity that can be used for the rapid diagnosis of IC, detect drug-resistant strains, and guide discontinuation of therapy.
- The development of antifungal drugs and regimens that do not require dose adjustment in patients with altered pharmacokinetics, such as those patients encountered within the ICU.
